# Excessively Enlarged Mitochondria in the Kidneys of Diabetic Nephropathy

**DOI:** 10.3390/antiox10050741

**Published:** 2021-05-07

**Authors:** Kiyoung Kim, Eun-Young Lee

**Affiliations:** 1 Department of Medical Biotechnology, Soonchunhyang University, Asan 31538, Korea; 2 Department of Medical Sciences, Soonchunhyang University, Asan 31538, Korea; 3 Division of Nephrology, Department of Internal Medicine, Soonchunhyang University Cheonan Hospital, Cheonan 31151, Korea; 4 Institute of Tissue Regeneration, College of Medicine, Soonchunhyang University, Cheonan 31151, Korea; 5 BK21 FOUR Project, College of Medicine, Soonchunhyang University, Cheonan 31151, Korea

**Keywords:** mitochondrial dynamics, oxidative stress, apoptosis, renal injury, diabetic nephropathy

## Abstract

Diabetic nephropathy (DN) is the most serious complication of diabetes and a leading cause of kidney failure and mortality in patients with diabetes. However, the exact pathogenic mechanisms involved are poorly understood. Impaired mitochondrial function and accumulation of damaged mitochondria due to increased imbalance in mitochondrial dynamics are known to be involved in the development and progression of DN. Accumulating evidence suggests that aberrant mitochondrial fission is involved in the progression of DN. Conversely, studies linking excessively enlarged mitochondria to DN pathogenesis are emerging. In this review, we summarize the current concepts of imbalanced mitochondrial dynamics and their molecular aspects in various experimental models of DN. We discuss the recent evidence of enlarged mitochondria in the kidneys of DN and examine the possibility of a therapeutic application targeting mitochondrial dynamics in DN.

## 1. Introduction

Diabetic nephropathy (DN) is the most common cause of end-stage kidney disease associated with diabetes mellitus (DM) and is one of the most serious complications of diabetes [1,2]. DN develops in approximately 30% of patients with type 1 DM and 40% of those with type 2 DM [1]. Podocyte loss, albuminuria, and a reduction in glomerular filtration rate are the major clinical features of DN [3,4]. DN also has significant long-term effects on the mortality of patients with diabetes [2]. Although several studies have been performed over a long period, the molecular mechanisms of DN pathogenesis have not been elucidated and there are no effective therapeutics for DN. Therefore, novel studies are needed to better understand the pathogenesis of DN and the exact molecular mechanisms involved to improve the pathological and clinical changes in the kidney. Recently, increasing evidence has shown that mitochondrial dysfunction, including imbalanced mitochondrial dynamics and excessive reactive oxygen species (ROS) production, plays an important role in the development and progression of DN.

Mitochondria play an essential role in the survival of all cells responsible for cellular respiration, ROS generation, and adenosine triphosphate (ATP) production by oxidative phosphorylation. The kidneys are highly energy-demanding tissues in the body and are rich in mitochondria. Thus, mitochondrial and kidney functions are highly linked. Proximal tubular cells in renal tissue are rich in mitochondria and rely on oxidative phosphorylation for ATP generation [5]. Moreover, the importance of mitochondrial function in the kidneys is evident in inherited mitochondrial diseases with renal impairment [6]. Previously, it has been reported that the mitochondrial membrane potential in endothelial cells and podocytes of the kidneys is reduced by treating high-glucose in DN [7]. Highly increased ROS production by mitochondria plays a role in metabolic and cellular signaling pathways, including inflammation and apoptosis, in response to hyperglycemia and hyperlipidemia [8,9,10]. In addition, accumulating evidence suggests that mitochondrial morphological changes, including fragmentation and enlargement induced by diabetic conditions such as hyperglycemia, play essential pathological roles in DN pathogenesis. In various DN experimental models, increased mitochondrial fission has been shown to be a key mediator of increased ROS production and apoptosis under hyperglycemic conditions [11]. However, recent research has found that excessive mitochondrial fusion can also be involved in DN pathogenesis [12,13]. Therefore, it is unclear how various mitochondrial morphologies are associated with mitochondrial function and which morphology is critical for inducing mitochondrial dysfunction, such as ROS production in DN. This means that dysfunctional mitochondria, such as fragmented or enlarged mitochondria, are increasingly postulated to be crucial to the development and progression of DN.

In this review, we provide an update on the recent advances in the relationship between mitochondrial dynamics and the progression of DN and an overview of the pathophysiological role of mitochondrial dynamics in the kidneys. We focus on evidence of aberrant mitochondrial morphological changes in DN and summarize the experimental studies supporting fragmented or enlarged mitochondria as the cause of DN both in vitro and in vivo. Moreover, we discuss in detail the role of mitochondrial enlargement in the development and progression of DN. In addition, we highlight potential therapeutic drugs and strategies that regulate mitochondrial dynamics and could potentially be beneficial in the treatment of DN.

## 2. Mitochondrial Dynamics and Oxidative Stress

### 2.1. Regulation of Mitochondrial Dynamics

Mitochondria are highly dynamic cellular organelles that constantly alter their shape and size through fission and fusion events [14]. This dynamic process allows the maintenance of the proper mitochondrial network in cells under various environments. Fission results in mitochondrial fragmentation, whereas fusion results in elongated and enlarged mitochondria. A balance between fission and fusion processes is required for the maintenance of optimal mitochondrial conditions because aberrant and imbalanced mitochondrial dynamics could finally result in mitochondrial dysfunction that induces intracellular stress or damage, including ROS production and apoptosis [15,16].

The regulation machinery of mitochondrial dynamics has been identified in yeast and *Drosophila* models, and homolog proteins have also been found in mammalian models [17,18]. These include the fusion regulation factors, mitofusin 1 and 2 (Mfn1/2) and optic atrophy 1 (Opa1), and the fission regulation factors, dynamin-related protein 1 (Drp1) and mitochondrial fission protein 1 (Fis1) [18].

Drp1, a large dynamin-related GTPase, translocalizes from the cytosol to the mitochondrial outer membrane (MOM) and forms a ring-like oligomeric structure around the membrane to be fragmented. After mitochondrial fission, Drp1 returns to the cytosol from the MOM. The binding of Drp1 to MOM is regulated by various receptors, including Fis1, mitochondrial fission factor (Mff), and mitochondrial dynamics proteins of 49 and 51 kDa (MiD49 and MiD51, respectively) [19]. The activation and translocation of Drp1 to mitochondria is regulated by posttranslational modifications, such as phosphorylation, ubiquitination, sumoylation, and nitrosylation [20]. Several kinases can influence Drp1 localization between the cytosol and mitochondria by phosphorylating two serine residues of Drp1 [20]. Downregulation of Drp1 expression leads to enhanced mitochondrial fusion, forming enlarged mitochondria, and overexpression of Drp1 induces mitochondrial fragmentation.

Fis1 is also a key factor in the regulation of mitochondrial dynamics and the receptor for Drp1 [21]. Fis1, a small membrane protein, is localized in the MOM through a single transmembrane domain at the C-terminus. Fis1 plays a major role in Drp1 localization from the cytosol to mitochondrial fission sites by physically binding to Drp1 [22]. Fis1 overexpression induces mitochondrial fission, resulting in fragmentation and apoptosis through the recruitment of Drp1. In addition, Fis1 knockdown by microRNA leads to mitochondrial fusion and elongation [21].

Mfn1 and Mfn2 are large mitochondrial transmembrane GTPases that belong to the dynamin superfamily, which can interact with both the homodimer (Mfn1-Mfn1 or Mfn2-Mfn2) and heterodimers (Mfn1-Mfn2) to mediate mitochondrial fusion [23]. Mfn1 and Mfn2 play a central role in the tethering and fusion of MOMs. These mitofusin complexes on adjacent mitochondrial membranes act as membrane tethers and form dimeric structures between each mitochondrial membrane [24]. When mitofusins are tethered, GTP hydrolysis enables mitochondrial fusion [25]. Mfn1- or Mfn2-deficient mutant cells are defective in mitochondrial function by inducing mitochondrial fragmentation, and they also exhibit several mitochondrial and cellular defects, including aberrant mitochondrial membrane potential, decreased respiration, excessive ROS production, and apoptosis [24,26]. Moreover, Mfn2 is involved in tethering mitochondria to the endoplasmic reticulum (ER) [27,28]. Therefore, Mfn1 and Mfn2 may have different functions in mitochondrial dynamics and interactions with other organelles.

Opa1 is also a mitochondrial dynamin-related GTPase anchored to the mitochondrial inner membrane (MIM) and plays an important role in mitochondrial fusion [29]. The *Opa1* gene is alternatively spliced and yields eight mRNAs. These variants are proteolytically processed into the inner membrane-anchored long-form of Opa1 (L-Opa1) and soluble short form of Opa1 (S-Opa1) [30]. L-Opa1 is only required for MIM fusion, but S-Opa1 is not [31]. In addition, Opa1 controls cristae morphogenesis in mitochondria [32,33]. Opa1 is also linked to mitochondrial oxidative phosphorylation in the respiratory chain and membrane potential through sequestering soluble cytochrome *c* within the cristae [32]. *Opa1* mutation in mice and *Drosophila* showed embryonic lethality, which indicates a critical function of mitochondrial fusion during development [34,35]. In both human and mouse models, *Opa1* mutation leads to abnormal mitochondrial morphology and mitochondrial dysfunction, including mitochondrial oxidative phosphorylation defects and mitochondrial DNA instability [36].

The pathogenic mechanism of imbalanced mitochondrial dynamics during the development and progression of several metabolic diseases, including diabetes mellitus, renal diseases, heart disease, and cancers, is currently being investigated. Aberrant mitochondrial dynamics may be complicated by mitochondrial fission and fusion processes. However, the molecular mechanisms leading to imbalanced mitochondrial dynamics and novel regulators of the modulation of mitochondrial morphology are largely unknown. Furthermore, it is unclear how increased mitochondrial fission or fusion status modulates cellular toxicity in diseases and whether the involvement of their imbalanced status is direct or indirect in the development of diseases. Therefore, further research is required to provide evidence on the relationships between mitochondrial dynamics, mitochondrial dysfunction, excessive ROS generation, and apoptosis.

### 2.2. Generation of Reactive Oxygen Species Due to Aberrant Mitochondrial Dynamics

ROS are reactive molecules and free radicals derived from molecular oxygen. ROS have an intracellular function at low levels and play the role of signaling molecules when strictly regulated [37]. Mitochondria are the main source of ROS in cellular systems. High levels of mitochondrial ROS can induce cellular stress and damage. Mitochondrial ROS are scavenged by various enzymes, including manganese superoxide dismutase (MnSOD) [38,39]. Enhanced mitochondrial ROS production has been implicated in the pathogenesis of various diseases, such as cancer, neurodegenerative diseases, and metabolic diseases, even with aging. Recently, it has been reported that overproduction of mitochondrial ROS is significantly associated with cellular damage in the kidneys and the progression of kidney disease. Hyperglycemia and hyperlipidemia are well-known factors that contribute to the development of DN [40,41]. It is also important to note that oxidative stress is linked to mitochondrial function, not just because mitochondria generate ROS, but also because ROS can cause deterioration of mitochondrial function.

Mitochondria can change various morphologies from small, short shapes to enlarged, elongated shapes. Recent studies have reported a relationship between mitochondrial fission and ROS production in various models. Increased mitochondrial fragmentation in Drp1 overexpression or Mfn1 knockdown in cultured human hepatocellular carcinoma cells is associated with excessive ROS production and apoptosis [42,43]. Mff overexpression in cultured human breast cancer cells has been reported to induce mitochondrial fission, mitochondrial membrane potential loss, and excessive ROS generation [44]. Moreover, Mfn1- and Mfn2-deficient cultured cells and macrophages of Mfn2 knockout mice showed mitochondrial fragmentation and increased ROS production [45,46,47]. These results suggest that increased mitochondrial fragmentation through modulation of mitochondrial dynamics machinery may lead to excessive ROS generation from mitochondria and can be associated with diseases.

However, excessive mitochondrial fusion, which results in elongated and enlarged mitochondria, also induces ROS production and cytotoxicity. Aged human hepatocytes, which display elongated mitochondria, have decreased mitochondrial bioenergetic capacity [48,49]. Increased mitochondrial elongation in Fis1 knockdown cultured cells has shown senescence-associated phenotypes, including increased DNA damage, ROS production, and loss of mitochondrial membrane potential [50]. Moreover, mitochondrial E3 ubiquitin ligase, membrane-associated RING-CH5 (MARCH5) knockdown in cultured cells promotes highly connected and elongated mitochondria, increased ROS levels, and reduced mitochondrial membrane potential by increasing Mfn1 expression and blocking Drp1 activity [51]. These results indicate that increased mitochondrial fusion may lead to stress-induced cellular senescence.

The presence of fragmented mitochondria owing to increased fission or decreased fusion can lead to mitochondrial dysfunction, including excessive ROS generation and decreased mitochondrial biogenesis [52,53]. Conversely, the increased formation of enlarged mitochondria owing to fusion activation or fission inhibition can diminish mitochondrial turnover by impairing mitophagy, leading to the accumulation of damaged mitochondria in cells [54,55]. The balance between mitochondrial fission and fusion to maintain mitochondrial function is an intricate cellular process that is still under investigation. Therefore, a critical question in the regulation of mitochondrial dynamics that leads to ROS production or cellular toxicity is how the expression of mitochondrial fission and/or fusion proteins is controlled and which morphological process is important in the development of the disease under various physiological or pathophysiological conditions.

## 3. Recent Advances on Mitochondrial Fission in Diabetic Nephropathy

### 3.1. In Vivo Studies Using Animal Models

Various in vitro studies have also implicated mitochondrial fission as a key mediator of increased ROS production and apoptosis under hyperglycemic conditions [11,56]. Hyperglycemia leads to renal injury in kidney tissues and induces DN. Therefore, the precise role of mitochondrial dynamics and novel regulators in DN pathogenesis needs to be investigated. In particular, the novel regulatory mechanisms of mitochondrial dysfunction, including aberrant morphological changes associated with mitochondrial ROS production under hyperglycemia in vivo DN models, need to be explored.

Many experiments have been conducted to investigate mitochondrial morphological changes in experimental models of DN (Table 1). Excessive mitochondrial fragmentation has been observed and identified as a pathogenic mechanism of diabetic nephropathy in various in vivo models. In 2012, researchers attempted to examine the role of mitochondrial dynamics in the progression of diabetic nephropathy using an in vivo mouse model [57]. They showed that albuminuria in diabetic models is reduced in ROCK1 knockout mice and that ROCK1 triggers mitochondrial fragmentation by phosphorylating Drp1 at Serine 600 [57]. Drp1 is translocalized to the outer membrane of mitochondria when it is activated by phosphorylation of serine residue, leading to a reduction in ATP levels in renal tubular cells [58]. In the podocytes of diabetic mice, mitochondrial fission is apparently active; deleting podocyte-specific Drp1 using conditional knockout strategy results in significantly decreased mitochondrial division, decreased proteinuria, and improved podocyte morphology [59]. Therefore, these studies demonstrate that hyperglycemia-induced mitochondrial fission is mediated by ROCK1 activation and Drp1 phosphorylation in mouse podocytes [57]. Furthermore, in another study, Galvan et al. generated a knock-in mouse in which S600 of Drp1 was mutated to alanine (Drp1S600A) and showed that Drp S600A transgenic mice with *db/db* background exhibited decreased progression of diabetic nephropathy, decreased mitochondrial ROS production, and mitochondrial fragmentation in kidney tissues [60]. Collectively, these findings suggest that Drp1 phosphorylation and translocalization to mitochondria by metabolic stress, including hyperglycemia, contribute to aberrant mitochondrial dynamics and renal dysfunction in the kidneys of diabetic nephropathy.

In addition, there is emerging in vivo evidence that mitochondrial damage in kidney tissues with diabetes is important for the pathogenesis of diabetic nephropathy. Several novel regulators have been found to participate in mitochondrial dysfunction, including ROS production and impaired mitochondrial dynamics induced by metabolic stress conditions, such as obesity, insulin resistance, and diabetes mellitus. Hyperglycemia induces the downregulation of peroxisome proliferator-activated receptor-γ coactivator-1α (PGC-1α), a regulator of oxidative metabolism in mitochondria, in the kidneys of diabetic rats [61]. Researchers have shown that decreased PGC-1α expression induces mitochondrial ROS production and mitochondrial fragmentation in the kidneys of high-dose glucose diet-fed rats. A type 2 diabetic animal model, *db/db* mice, also showed renal injury and increased Drp1 expression in the kidneys [62]. Moreover, apoptosis-inducing factor (AIF) is a mitochondrial flavoprotein with dual roles in redox signaling and programmed cell death. AIF expression is significantly decreased in the renal tubules of patients with CKD [63]. AIF knockdown mice with diabetes have shown severe kidney injury, including glomerular damage, mitochondrial dysfunction, and fragmentation. This phenotype is also shown to be regulated by the increased localization of Drp1 to the renal mitochondria in AIF knockout mice with diabetes.

Myo-inositol oxygenase (MIOX), which catabolizes myo-inositol to D-glucuronate, regulates apoptosis in tubular cells of diabetes [64]. A previous report found that MIOX expression and activity were increased in kidney cells of *db/db* mice [65]. Activated MIOX during hyperglycemia induces mitochondrial fission and dysfunction in streptozotocin (STZ)-induced diabetic mice [66]. Interestingly, dietary treatment with MIOX inhibitor in diabetic mice decreased apoptosis, increased mitophagy, and improved renal function.

Dual-specificity protein phosphatase-1 (DUSP1) is a threonine/tyrosine phosphatase that dephosphorylates p38 and c-Jun N-terminal kinase (JUN) [67]. DUSP1 is downregulated in the renal tissue of hyperglycemia-induced diabetic mice. Overexpression of DUSP1 in diabetic mice attenuates renal dysfunction and diabetic kidney damage [68]. DUSP1 overexpression suppresses hyperglycemia-induced mitochondrial fission by regulating Mff-related mitochondrial dynamics. In addition, downregulated DUSP1 expression induced by hyperglycemia activates JNK pathway, leading to phosphorylated Mff-mediated mitochondrial fission [68].

p66Shc is a member of the Src homologous-collagen homolog (ShcA) adaptor protein family [69]. It has been regarded as a key regulator of mitochondrial ROS production and apoptosis, and is involved in several diseases, including metabolic diseases. The expression of p66Shc and phosphorylated p66Shc was highly increased in the kidney tissues of both STZ-induced diabetic mice and *db/db* mice [70]. p66Shc knockdown alleviated increased mitochondrial fragmentation, downregulated Fis1, reduced p66Shc-Fis1 binding, and increased Mfn1 expression under high-glucose conditions [71].

Nuclear receptor subfamily 4 group A member 1 (NR4A1) is activated in response to hyperglycemia and contributes to the pathogenesis of diabetic nephropathy. Genetic depletion of NR4A1 suppresses glomerular apoptosis, renal dysfunction, and mitochondrial damage induced by a high-glucose diet. Furthermore, NR4A1 modulates Mff and parkin expression via the p53 signaling pathway. NR4A1 knockdown suppresses high-glucose-induced mitochondrial fragmentation by modulating Miff and parkin transcription and improves diabetic nephropathy [72].

Metabolic disorders caused by oxidative stress are believed to be prominent in renal injury during the progression of diabetic nephropathy, resulting in hypoxia. It is well known that hypoxia-inducible factor-1 (HIF-1) is a key regulator of hypoxic stress and damage. HIF-1 is a heterodimeric protein consisting of two subunits, HIF-1α and HIF-1β [73]. HIF-1α is degraded by the proteasome under normoxic conditions and is stabilized under hypoxic conditions [74]. The expression of HIF-1α is increased in diabetic mice. Conditional deletion of HIF-1α in proximal tubular cells of STZ-induced diabetic mice enhances kidney injury and mitochondrial fission by increasing Drp1, phosphorylated Drp-1, and Fis1 [75]. Moreover, HIF-1α regulates heme oxygenase-1 (HO-1) expression directly [75]. This result suggests that HIF-1α improved mitochondrial dysfunction and apoptosis in tubular cells of DN via the HO-1 pathway. Collectively, there is sufficient in vivo evidence to support the role of regulators of mitochondrial dynamics in the development and progression of diabetic nephropathy. 

Renal diseases, including acute kidney injury (AKI) and chronic kidney disease (CKD), are characterized by renal tissue damage. AKI is a major kidney disease that contributes to the development of CKD. Previous studies have shown that mitochondrial pathology has been implicated in AKI [76]. Funk et al. found that renal injury, apoptosis, and mitochondrial dysfunction in glycerol-injected acute kidney injury models were associated with mitochondrial fragmentation [77]. Both Drp1 and Mfn2 protein expression were markedly elevated after glycerol injection. This result suggests that persistent mitochondrial dysfunction occurs in the proximal tubular cells of the kidney tissues after acute kidney stress. Sepsis is the most common risk factor for AKI. Liu et al. generated a septic AKI mouse model by inducing cecal ligation and puncture (GLP) [78]. The expression of Opa1 was decreased, while that of Drp1 was increased in the kidneys after CLP, indicating that mitochondrial fusion was inhibited and fission was enhanced. These findings demonstrate that aberrant mitochondrial dynamics contribute to renal injury in AKI and provide further support for the pathophysiological relationship between AKI, CKD, and DN, which are associated with mitochondrial dynamics.

Accumulating evidence suggests that further research on aberrant mitochondrial dynamics balance between fission and fusion induced by diabetic conditions could facilitate the development of a potential therapeutic strategy for DN.

### 3.2. In Vitro Studies Using Cell Models

Consistent with animal studies, in vitro studies using various kidney cells also provide evidence that hyperglycemia activates various pathways and regulators that lead to mitochondrial dynamic imbalance. Wang et al. found that Rho-associated coiled coil-containing protein kinase 1 (ROCK1) mediates mitochondrial fragmentation induced by high glucose treatment in cultured mouse podocytes [57]. PGC-1α inhibits hyperglycemia-induced excessive ROS production and mitochondrial fragmentation in rat glomerular mesangial cells [61]. High glucose treatment leads to increased mitochondrial fission, mitochondrial ROS production, and apoptosis in renal proximal tubular cells [66]. Inhibition of MIOX using shRNA in HK-2 cells suppresses mitochondrial fragmentation, ROS production, and Drp1 expression under high glucose treatment. p66Shc modulates mitochondrial dynamics, ROS production, and apoptosis in renal tubular cells under high-glucose conditions [71]. This in vitro study demonstrates a regulatory role of p66Shc in hyperglycemia-induced mitochondrial fragmentation through modulation of the mitochondrial proteins Fis1 and Mfn1 [71]. Hypoxia induces the expression of HIF-1α and mitochondrial fission and enhances mitochondrial membrane potential loss, ROS production, and apoptosis in HK-2 cells. HIF-1α-mediated mitochondrial morphological changes under hypoxic conditions are regulated by HO-1 in HK-2 cells [75].

**Table 1:** Regulators that affect mitochondrial dynamics in various experimental models of diabetic nephropathy.

Experimental Models: In Vivo and In Vitro	Phenotypes on Mitochondria	Regulator for Mitochondrial Dynamics in Diabetic Nephropathy Progression	References
*db/db* mice STZ-treated mice Podocyte Glomerular endothelial cell	Increased mitochondrial fission Increased Drp1 expression Increased p-Drp1 at Ser600	Rho-associated coiled coil-containing protein kinase 1 (ROCK1) *ROCK1* deletion: Suppression of renal injury	[57]
STZ-treated rat Rat glomerular mesangial cell Human proximal tubular cell	Increased mitochondrial fission Increased ROS production Increased Drp1 expression Decreased Mfn1 expression	Peroxisome proliferator-activated receptor-γ coactivator-1α (PGC-1α) *PGC-1α* knockdown: Suppression of renal injury	[61,79]
STZ-treated mice Human proximal tubular cell	Increased mitochondrial fission Decreased mitochondrial membrane potential Increased ROS production Increased apoptosis Increased Drp1, Fis1 expression Decreased Mfn2 expression	Myo-inositol oxygenase (MIOX) MIOX overexpression: Suppression of renal injury	[66]
STZ-treated mice Human renal mesangial cell	Increased mitochondrial fission Decreased mitochondrial membrane potential Increased ROS production Increased cytochrome c release Increased apoptosis Increased Drp1, Mff expression Decreased Mfn1, Opa1 expression	Dual-specificity protein phosphatase-1 (DUSP1) DUSP1 overexpression: Suppression of renal injury	[68]
Human DN Patient Human proximal tubular cell	Increased mitochondrial fission Decreased mitochondrial membrane potential Increased ROS production Increased cytochrome c release Increased apoptosis Increased Drp1, Fis1 expression Decreased Mfn1 expression	p66 Src homologous-collagen homologue (p66Shc) *p66Shc* knockdown: Suppression of renal injury	[71]
STZ-treated mice Human renal mesangial cell	Increased mitochondrial fission Decreased mitochondrial membrane potential Increased ROS production Increased cytochrome c release Increased apoptosis Increased Drp1, Mff expression Decreased Mfn1 expression	Nuclear receptor subfamily 4 group A member 1 (NR4A1) *NR4A1* deletion: Suppression of renal injury	[72]
STZ-treated mice Human proximal tubular cell	Increased mitochondrial fission Decreased mitochondrial membrane potential Increased ROS production Increased cytochrome c release Increased apoptosis Increased Drp1, Fis1 expression Increased p-Drp1 level Decreased Mfn1, Mfn2 expression	Hypoxia-inducible factor-1α (HIF-1α) *HIF-1α* deletion: Enhancement of renal injury	[75]
Human proximal tubular cell	Increased mitochondrial fusion Decreased mitochondrial respiration Decreased ROS-induced apoptosis Decreased ATP interaction with Mfn1, Mfn2	Induced in high glucose-1 (IHG-1) IHG-1 overexpression: Protection from apoptosis in kidney cells	[80]
Human DN Patient Harlequin mice Human proximal tubular cell	Increased mitochondrial fusion Increased Mfn1, Mfn2 expression Increased Opa1 expression	Apoptosis-inducing factor (AIF) *AIF* knockdown: Display renal injury	[63]

### 3.3. Clinical Evidence from Human Studies

Mitochondrial fission has been detected in the kidneys of patients with diabetes. Highly increased mitochondrial fragmentation and loss of mitochondrial networks have been observed in isolated endothelial cells from patients. Furthermore, increased expression of Drp1 and Fis1 has been observed in freshly isolated endothelial cells from patients with diabetes mellitus [81]. In addition, Zhan et al. observed changes in mitochondrial morphology and the expression of mitochondrial dynamic-associated proteins in renal proximal tubular cells from patients with diabetic nephropathy [71]. They showed that aberrant mitochondrial dynamics modulated by p66Shc led to increased ROS production and apoptosis [71]. Thus, this result suggests that mitochondrial fragmentation is a critical feature of renal injury that is associated with oxidative stress and p66Shc phosphorylation. Moreover, fragmented mitochondria were dominant in podocytes and proximal tubular cells of patients with DN [82]. Drp1 localization in the mitochondrial outer membrane was increased in the glomeruli of patients with DN. Consistent with previous studies, these findings suggest that the mitochondrial dynamics-associated protein Drp1 is involved in podocyte injury in DN, and increased mitochondrial fission may contribute to podocyte dysfunction in diabetic conditions.

## 4. Recent Studies for Enlarged and Highly Fused Mitochondria in Diabetic Nephropathy

Excessive mitochondrial fusion, like excessive mitochondrial fission, can be involved in disease pathogenesis, as seen in various neurodegenerative diseases, including Parkinson’s disease [83]. Previous studies have described enlarged mitochondrial formation in several human diseases, including diabetes, acute glomerulonephritis, and alcoholic cardiomyopathy [84,85,86]. In addition, giant mitochondria may contribute to cellular senescence in animal and plant cells [49,87]. Sustained elongation of mitochondria is associated with cellular senescence, decreased mitochondrial membrane potential, increased reactive oxygen species production, and DNA damage [50]. Although evidence for the effects of enlarged mitochondria in DN pathogenesis is insufficient, recent advanced studies have been carried out to investigate whether mitochondrial morphological defects or changes can lead to the development and progression of DN.

Induced in high glucose-1 (IHG-1), which is known as tRNA-histidine guanylyl transferase 1-like, was identified as a glucose-regulated transcript associated with DN [88,89]. A previous study showed that IHG-1 expression is increased in human DN [90]. Hickey et al. showed that IHG-1 overexpression increases mitochondrial fusion by physically interacting with the mitochondrial fusion proteins Mfn1 and Mfn2 in HeLa cells and HEK293T cells [80]. Moreover, the physical interaction between IHG-1 and Mfn is necessary for increased IHG-1-mediated mitochondrial fusion. Increased HIG-1 levels protect cells from ROS-induced apoptosis [80]. Thus, they proposed that increased IHG-1 expression in DN protects cell viability in renal tissues.

AIF is a caspase-independent cell death effector [91]. Previous studies have shown that AIF regulates the stability, assembly, and activity of complex I in mitochondria [92]. Coughlan et al. showed that the expression of AIF is significantly decreased in the renal tubules of human DN and investigated the role of AIF in kidney function in mice [63]. AIF knockdown led to renal hypertrophy and increased urinary albumin excretion. Interestingly, mitochondria in proximal tubular cells of AIF knockdown mice were more elongated and enlarged due to increased expression of Mfn1 and Mfn2, which are mitochondrial fusion proteins, in renal tissues [63]. However, when AIF was knocked down with diabetes, mitochondrial morphology was more fragmented [63]. These studies suggest that a deficiency in AIF results in changes in mitochondrial function, networking, and ROS production, which could contribute to renal disease. Under diabetic conditions, a switch from mitochondrial fusion to fission, impaired electron transport chain, and ATP depletion leads to more advanced renal injury. Thus, this result supports a critical role for AIF in the maintenance of renal function through the modulation of mitochondrial homeostasis. Aberrant mitochondrial dynamics and morphological changes, including switching fusion to fission in the progression of DN, should be investigated in further studies.

In addition, Woo et al. unexpectedly observed enlarged mitochondria in the podocytes of Otsuka Long-Evans Tokushima Fatty (OLETF) rat models [12]. The OLETF rat is a well-known animal model of obesity and type 2 diabetes. OLETF rats showed increased urinary albumin excretion and elongated mitochondria in the kidney podocytes. Moreover, to investigate the changes in mitochondrial morphology, they also used STZ-induced diabetic rats and *db/db* mice. Consistent with other studies, albuminuria was increased in STZ-induced rats and *db/db* mice. Interestingly, enlarged and elongated mitochondria were prominent features in both STZ-induced diabetic rats and *db/db* mouse models [12]. Furthermore, glucose, fatty acids, and angiotensin II (GFA)-combined treatment of cultured podocytes, which are used as DN cellular models, revealed that apoptosis, mitochondrial elongation, and excessive ROS production were increased, and autophagy was decreased [12]. These results suggest that damaged mitochondria from various metabolic stresses, such as high levels of glucose and fatty acids, are not removed by mitochondrial autophagy and mitophagy, leading to the formation of excessively enlarged mitochondria in kidney tissues.

Recently, we also found enlarged mitochondria in other experimental models of DN. Abnormality and dysfunction of *Drosophila* nephrocytes, which are podocyte-like cells, were found to be associated with a chronic high-sucrose diet. Moreover, we found chronic high-sucrose diet-induced excessive mitochondrial fusion in podocyte-like cells of *Drosophila* by upregulating the expression of mitochondrial fusion proteins Opa1 and Marf (homologous to human Mfn2) [13]. Our study revealed that a chronic high-sucrose diet induces morphological abnormalities in podocyte-like cells, as well as altered expression of Marf and Opa1, leading to disrupted and enlarged mitochondria in podocyte-like cells using an in vivo *Drosophila* model. Therefore, modulating the activity of fusion proteins might have an effect on the interaction between the mitochondrial network and cellular toxicity.

The above results suggest that mitochondrial enlargement observed in DN is associated with the renal injury caused by mitochondrial dysfunction in the kidney. However, it seems still not firmly established whether enlarged mitochondria perform pathophysiologically worse or better than fragmented ones in kidney tissues of DN. Ongoing studies are being carried out to understand the mechanisms that induce excessive enlargement of the mitochondria for the maintenance of kidney function under diabetic conditions. Further work is required to identify novel regulators and cellular pathways involved in triggering mitochondrial hyperfusion and toxicity in the kidney.

## 5. Pharmacological Modulation of Mitochondrial Dynamics

There are several pharmacological treatments for modulating the balance of mitochondrial dynamics in kidney injury (Table 2). Ayanga et al. investigated whether inhibition of Drp1 activity could rescue defective phenotypes, including renal injury, in a DN model [59]. Treatment of cultured podocytes with Midivi1, an inhibitor of Drp1 GTPase activity, significantly reduced high glucose-induced Drp-1 Ser600 phosphorylation and mitochondrial fission. Consistent with the cell culture model, inhibition of Drp1 activity by treating Midivi1 suppressed renal injury, including podocyte number, podocyte foot process, and mitochondrial fission in *db/db* mice with diabetes. These results suggest that the inhibition of mitochondrial fission improved the progression of DN.

Hyperglycemia induced downregulation of PGC-1α in the kidney of diabetic rats [61]. Reduction of PGC-1α expression led to excessive mitochondrial ROS production and mitochondrial fragmentation in the kidney of high-dose glucose diet-fed rats [10]. Lee et al. investigated the effect of PGC-1α activators, 5-aminoimidazole-4-carboxamide ribonucleotide (AICAR) and metformin, on mitochondrial fusion, fission, and autophagic quality control in renal proximal tubular cells under diabetic conditions [79]. AICAR or metformin reduces increased ROS production and apoptosis in high glucose-treated proximal tubular cells. Moreover, AICAR or metformin treatment suppresses albuminuria, renal dysfunction, increased Drp1 expression, and mitochondrial fission in STZ-induced diabetic mice. In addition, chloroquine (CQ) and amodiaquine (AQ) treatments, which are used as anti-malarial drugs and AMP-activated protein kinase (AMPK) activators, increase PGC-1α phosphorylation in proximal tubular cells under high-glucose conditions [93]. CQ and AQ treatment alleviates excessive ROS production, apoptosis, and mitochondrial fragmentation in high glucose-induced proximal tubular cells. CQ and AQ also ameliorate albuminuria and renal morphological defects in the kidneys of STZ-induced mice with diabetes [94]. 

Dipeptidyl peptidase-4 (DPP-4) inhibitors exert hypoglycemic effects by inhibiting the degradation of glucagon-like peptide-1 (GLP-1) in patients with type 2 diabetes mellitus [95]. Recent evidence has revealed that DPP-4 inhibitors such as saxagliptin and sitagliptin may exhibit a protective effect against the progression of renal injuries in diabetes. Zhang et al. showed that a renal tubular injury in diabetic mice was suppressed by sitagliptin treatment. Moreover, sitagliptin restored mitochondrial fragmentation by regulating Drp1 expression and phosphorylation in the kidneys of diabetic mice and albumin-overloaded human kidney-2 (HK-2) cells [96,97]. In addition, the β_2_-adrenergic receptor (AR) agonist formoterol induced mitochondrial biogenesis and promoted recovery from AKI [98]. Cleveland et al. found that pharmacological activation of the β_2_-adrenergic receptor (AR) by formoterol promotes recovery from kidney injury by inducing mitochondrial biogenesis in high glucose-treated proximal tubular cells and diabetic *db/db* models [99].

In addition, some molecules directly regulate and stimulate mitochondrial biogenesis. SS31, a novel mitochondrial target peptide, prevented renal injury in STZ-induced mice by decreasing mitochondrial fragmentation by suppressing the expression of Drp1 and increasing the expression of Mfn1 [100]. Melatonin is also used as a mitochondria-targeting agent. Therefore, it could be an effective strategy to modulate defective mitochondrial dynamics and excessive ROS production and ameliorate renal injury in diabetes. Agil et al. found that melatonin improved renal function, including proteinuria, in Zücker Diabetic Fatty (ZDF) rat models and modulated their mitochondrial dynamics by reducing the expression of Drp1 and increasing that of Mfn2 and Opa1 [101].

Several readily available agents, including natural compounds from plants, have been explored for therapeutic benefits in DN. Liu et al. investigated the effect of astragaloside IV (AS-IV), a small molecular saponin found in *Astragalus membranaceus (Fisch) Bge*, on the progression of DN in diabetic mice models [62]. Although recent studies have shown that AS-IV administration ameliorates DN in STZ-induced diabetic rats, the mechanism by which AS-IV ameliorates DN progression remains unknown. They found that AS-IV downregulates mitochondrial fission proteins, including Drp-1 and Fis1, in the kidney tissues of *db/db* mice [62]. Thus, AS-IV restrains increased mitochondrial fragmentation in the kidney and rescued renal injury in DN mice. Polydatin (PD), a resveratrol glycoside extracted from *Polygonum cuspidatum*, has antioxidant and anti-inflammatory effects [102]. Previous studies have reported that PD suppresses fibronectin accumulation in glomerular mesangial cells and ameliorates renal function in diabetic rats [103]. Ni et al. showed that PD treatment attenuates apoptosis and ROS production in both renal tissues of mice and high glucose-induced podocyte models. Furthermore, PD treatment inhibits mitochondrial fission in high glucose-induced podocytes by suppressing Drp1 expression and phosphorylation [94]. Empagliflozin is a selective inhibitor of sodium-glucose cotransporter-2 (SGLT-2). Empagliflozin reduces ROS production and apoptosis in proximal tubular cells in a high-glucose environment. It also attenuates mitochondrial fission and enhances autophagy in renal tubular cells of STZ-induced diabetic mice [104,105]. Berberine (BBR) is a plant isoquinoline alkaloid that is widely used in herbal medicines for the treatment of diabetes mellitus (DM) [106]. BBR strongly inhibits apoptosis and increases ROS generation, mitochondrial fragmentation, and dysfunction in palmitic acid (PA)-induced mouse podocytes. BBR can stabilize mitochondrial morphology in podocytes by abolishing PA-induced activation of Drp1 in both podocytes with PA exposure and in *db/db* mice [107]. Collectively, most experimental pharmacological treatments are believed to modulate aberrant mitochondrial dynamics, such as increased fission during DN progression. Nonetheless, further research is required to confirm the beneficial effects of these pharmacological treatments on impaired renal function.

**Table 2:** Studies on the physiological function of pharmacological agents in diabetic nephropathy.

Pharmacological Agents	Diabetic Nephropathy Models	Phenotypes	References
Midivi1 (Drp1 inhibitor)	*db/db* mice Mouse podocyte	Decreased p-Drp1 expression Inhibition of mitochondrial fragmentation Decreased ROS production Suppression of renal injury	[59]
AICAR & Metformin (PGC-1γ activator)	Human proximal tubular cell	Inhibition of mitochondrial fragmentation Decreased apoptosis Decreased ROS production Suppression of renal injury	[79]
CQ & AQ (AMPK activator)	STZ-induced mice Human proximal tubular cell	Decreased Drp1 expression Increased Mfn1 expression Inhibition of mitochondrial fragmentation Decreased apoptosis Decreased ROS production Suppression of renal injury	[93]
Sitagliptin (DPP-4 inhibitor)	STZ-induced mice Human proximal tubular cell	Decreased Drp1, p-Drp1 expression Increased Mfn2, Opa1 expression Inhibition of mitochondrial fragmentation Recovery of mitochondrial membrane potential Decreased apoptosis Decreased cytochrome c release Decreased ROS production Suppression of renal injury	[96]
Formoterol (β_2_-AR agonist)	*db/db* mice Human proximal tubular cell	Decreased Drp1 expression Increased Mfn1, Mfn2 expression Increased ATP	[99]
SS31 (Antioxidant mitochondrial-targeted peptide)	STZ-induced mice Human proximal tubular cell	Decreased Drp1 expression Increased Mfn1 expression Inhibition of mitochondrial fragmentation Recovery of mitochondrial membrane potential Decreased apoptosis Decreased ROS production Suppression of renal injury	[100]
Melatonin (Mitochondrial-targeted agent)	ZDF rat	Decreased Drp1 expression Increased Mfn2, Opa1 expression Increased oxygen consumption Increased mitochondrial respiration Suppression of renal injury	[101]
Polydatin (Resveratrol glycoside)	KKAy mice Mouse podocyte	Decreased p-Drp1 expression Inhibition of mitochondrial fragmentation Decreased apoptosis Decreased ROS production Suppression of renal injury	[94]
Empagliflozin (SGLT-2 inhibitor)	STZ-induced mice KKAy mice Human proximal tubular cell	Decreased Drp1 expression Increased Mfn1 expression Inhibition of mitochondrial fragmentation Recovery of mitochondrial membrane potential Increased ATP Decreased cytochrome c release Decreased apoptosis Decreased ROS production Suppression of renal injury	[104,105]
Berberine (Isoquinoline alkaloid)	*db/db* mice Mouse podocyte	Decreased p-Drp1 expression Inhibition of mitochondrial fragmentation Recovery of mitochondrial membrane potential Increased ATP Decreased apoptosis Decreased ROS production Suppression of renal injury	[107]

## 6. Conclusions

Disruption of mitochondrial homeostasis can result in mitochondrial dysfunction and cellular damage. Although accumulating evidence suggests that mitochondrial dynamics are linked to the development and progression of DN, the precise role and mechanism of aberrant mitochondrial morphology in renal injury remain to be determined. Here, we summarized the experimental evidence supporting the role of imbalanced mitochondrial dynamics in the development of DN. Most previous studies have focused on the increased mitochondrial fission status, which results in DN progression. In addition, several pharmacological agents targeting the mitochondria and regulating mitochondrial dynamics have been studied. Conversely, reports of excessively enlarged mitochondrial status linked to DN pathogenesis are emerging. The mechanism may involve decreased fission, increased fusion, and impaired mitophagy, suggesting that fusion beyond its physiological limit may be detrimental to mitochondrial function that regulates disease conditions. Therefore, it is possible that preventing mitochondrial hyperfusion in the kidney could enhance mitochondrial health and reduce renal injury. However, the molecular mechanisms and pathways related to hyperglycemia-induced mitochondrial morphological changes are complicated, and the understanding of imbalanced mitochondrial dynamics in the kidney is still immature. Based on the current studies on excessively enlarged mitochondria in DN progression, effective pharmacological agents are needed that may enhance mitophagy to reduce damaged mitochondria. It may facilitate the development of a more specific and effective treatment.

## References

[1] Reutens A.T. (2013). Epidemiology of diabetic kidney disease. Med. Clin. North. Am..

[2] Chen Y., Lee K., Ni Z., He J.C. (2020). Diabetic Kidney Disease: Challenges, Advances, and Opportunities. Kidney Dis..

[3] White K.E., Bilous R.W., Marshall S.M., El Nahas M., Remuzzi G., Piras G., De Cosmo S., Viberti G. (2002). Podocyte number in normotensive type 1 diabetic patients with albuminuria. Diabetes.

[4] Toyoda M., Najafian B., Kim Y., Caramori M.L., Mauer M. (2007). Podocyte detachment and reduced glomerular capillary endothelial fenestration in human type 1 diabetic nephropathy. Diabetes.

[5] Che R., Yuan Y., Huang S., Zhang A. (2014). Mitochondrial dysfunction in the pathophysiology of renal diseases. Am. J. Physiol Ren. Physiol..

[6] Govers L.P., Toka H.R., Hariri A., Walsh S.B., Bockenhauer D. (2021). Mitochondrial DNA mutations in renal disease: An overview. Pediatra Nephrol..

[7] Srivastava S.P., Kanasaki K., Goodwin J.E. (2020). Loss of Mitochondrial Control Impacts Renal Health. Front. Pharm..

[8] Woo C.Y., Baek J.Y., Kim A.R., Hong C.H., Yoon J.E., Kim H.S., Yoo H.J., Park T.S., Kc R., Lee K.U. (2020). Inhibition of Ceramide Accumulation in Podocytes by Myriocin Prevents Diabetic Nephropathy. Diabetes Metab. J..

[9] Xu S., Nam S.M., Kim J.H., Das R., Choi S.K., Nguyen T.T., Quan X., Choi S.J., Chung C.H., Lee E.Y. (2015). Palmitate induces ER calcium depletion and apoptosis in mouse podocytes subsequent to mitochondrial oxidative stress. Cell Death Dis..

[10] Das R., Xu S., Nguyen T.T., Quan X., Choi S.K., Kim S.J., Lee E.Y., Cha S.K., Park K.S. (2015). Transforming Growth Factor beta1-induced Apoptosis in Podocytes via the Extracellular Signal-regulated Kinase-Mammalian Target of Rapamycin Complex 1-NADPH Oxidase 4 Axis. J. Biol. Chem..

[11] Rube D.A., van der Bliek A.M. (2004). Mitochondrial morphology is dynamic and varied. Mol. Cell Biochem..

[12] Woo C.Y., Kc R., Kim M., Kim H.S., Baek J.Y., Koh E.H. (2020). Autophagic flux defect in diabetic kidney disease results in megamitochondria formation in podocytes. Biochem. Biophys. Res. Commun..

[13] Kim K., Cha S.J., Choi H.J., Kang J.S., Lee E.Y. (2021). Dysfunction of Mitochondrial Dynamics in Drosophila Model of Diabetic Nephropathy. Life.

[14] Nunnari J., Suomalainen A. (2012). Mitochondria: In sickness and in health. Cell.

[15] Detmer S.A., Chan D.C. (2007). Functions and dysfunctions of mitochondrial dynamics. Nat. Rev. Mol. Cell Biol..

[16] Westermann B. (2010). Mitochondrial fusion and fission in cell life and death. Nat. Rev. Mol. Cell Biol..

[17] Okamoto K., Shaw J.M. (2005). Mitochondrial morphology and dynamics in yeast and multicellular eukaryotes. Annu. Rev. Genet..

[18] Saneto R.P. (2020). Mitochondrial diseases: Expanding the diagnosis in the era of genetic testing. J. Transl. Genet. Genom..

[19] Zhang Z., Liu L., Wu S., Xing D. (2016). Drp1, Mff, Fis1, and MiD51 are coordinated to mediate mitochondrial fission during UV irradiation-induced apoptosis. FASEB J..

[20] Adaniya S.M., O-Uchi J., Cypress M.W., Kusakari Y., Jhun B.S. (2019). Posttranslational modifications of mitochondrial fission and fusion proteins in cardiac physiology and pathophysiology. Am. J. Physiol. Cell Physiol..

[21] Lee Y.J., Jeong S.Y., Karbowski M., Smith C.L., Youle R.J. (2004). Roles of the mammalian mitochondrial fission and fusion mediators Fis1, Drp1, and Opa1 in apoptosis. Mol. Biol. Cell.

[22] James D.I., Parone P.A., Mattenberger Y., Martinou J.C. (2003). hFis1, a novel component of the mammalian mitochondrial fission machinery. J. Biol. Chem..

[23] Filadi R., Pendin D., Pizzo P. (2018). Mitofusin 2: From functions to disease. Cell Death Dis..

[24] Chen H., Detmer S.A., Ewald A.J., Griffin E.E., Fraser S.E., Chan D.C. (2003). Mitofusins Mfn1 and Mfn2 coordinately regulate mitochondrial fusion and are essential for embryonic development. J. Cell Biol..

[25] Ishihara N., Eura Y., Mihara K. (2004). Mitofusin 1 and 2 play distinct roles in mitochondrial fusion reactions via GTPase activity. J. Cell Sci..

[26] Chen H., Chomyn A., Chan D.C. (2005). Disruption of fusion results in mitochondrial heterogeneity and dysfunction. J. Biol. Chem..

[27] van Vliet A.R., Agostinis P. (2018). Mitochondria-Associated Membranes and ER Stress. Curr. Top. Microbiol. Immunol..

[28] Munoz J.P., Ivanova S., Sanchez-Wandelmer J., Martinez-Cristobal P., Noguera E., Sancho A., Diaz-Ramos A., Hernandez-Alvarez M.I., Sebastian D., Mauvezin C. (2013). Mfn2 modulates the UPR and mitochondrial function via repression of PERK. EMBO J..

[29] Delettre C., Lenaers G., Griffoin J.M., Gigarel N., Lorenzo C., Belenguer P., Pelloquin L., Grosgeorge J., Turc-Carel C., Perret E. (2000). Nuclear gene OPA1, encoding a mitochondrial dynamin-related protein, is mutated in dominant optic atrophy. Nat. Genet..

[30] Del Dotto V., Fogazza M., Carelli V., Rugolo M., Zanna C. (2018). Eight human OPA1 isoforms, long and short: What are they for?. Biochim. Biophys. Acta. Bioenerg..

[31] Song Z., Chen H., Fiket M., Alexander C., Chan D.C. (2007). OPA1 processing controls mitochondrial fusion and is regulated by mRNA splicing, membrane potential, and Yme1L. J. Cell Biol..

[32] Olichon A., Baricault L., Gas N., Guillou E., Valette A., Belenguer P., Lenaers G. (2003). Loss of OPA1 perturbates the mitochondrial inner membrane structure and integrity, leading to cytochrome c release and apoptosis. J. Biol. Chem..

[33] Frezza C., Cipolat S., Martins de Brito O., Micaroni M., Beznoussenko G.V., Rudka T., Bartoli D., Polishuck R.S., Danial N.N., De Strooper B. (2006). OPA1 controls apoptotic cristae remodeling independently from mitochondrial fusion. Cell.

[34] McQuibban G.A., Lee J.R., Zheng L., Juusola M., Freeman M. (2006). Normal mitochondrial dynamics requires rhomboid-7 and affects Drosophila lifespan and neuronal function. Curr. Biol..

[35] Alavi M.V., Bette S., Schimpf S., Schuettauf F., Schraermeyer U., Wehrl H.F., Ruttiger L., Beck S.C., Tonagel F., Pichler B.J. (2007). A splice site mutation in the murine Opa1 gene features pathology of autosomal dominant optic atrophy. Brain.

[36] Amati-Bonneau P., Valentino M.L., Reynier P., Gallardo M.E., Bornstein B., Boissiere A., Campos Y., Rivera H., de la Aleja J.G., Carroccia R. (2008). OPA1 mutations induce mitochondrial DNA instability and optic atrophy ‘plus’ phenotypes. Brain.

[37] Ray P.D., Huang B.W., Tsuji Y. (2012). Reactive oxygen species (ROS) homeostasis and redox regulation in cellular signaling. Cell Signal..

[38] Indo H.P., Yen H.C., Nakanishi I., Matsumoto K., Tamura M., Nagano Y., Matsui H., Gusev O., Cornette R., Okuda T. (2015). A mitochondrial superoxide theory for oxidative stress diseases and aging. J. Clin. Biochem. Nutr..

[39] Nishikawa T., Edelstein D., Du X.L., Yamagishi S., Matsumura T., Kaneda Y., Yorek M.A., Beebe D., Oates P.J., Hammes H.P. (2000). Normalizing mitochondrial superoxide production blocks three pathways of hyperglycaemic damage. Nature.

[40] Forbes J.M., Coughlan M.T., Cooper M.E. (2008). Oxidative stress as a major culprit in kidney disease in diabetes. Diabetes.

[41] Susztak K., Raff A.C., Schiffer M., Bottinger E.P. (2006). Glucose-induced reactive oxygen species cause apoptosis of podocytes and podocyte depletion at the onset of diabetic nephropathy. Diabetes.

[42] Jezek J., Cooper K.F., Strich R. (2018). Reactive Oxygen Species and Mitochondrial Dynamics: The Yin and Yang of Mitochondrial Dysfunction and Cancer Progression. Antioxidants.

[43] Huang Q., Zhan L., Cao H., Li J., Lyu Y., Guo X., Zhang J., Ji L., Ren T., An J. (2016). Increased mitochondrial fission promotes autophagy and hepatocellular carcinoma cell survival through the ROS-modulated coordinated regulation of the NFKB and TP53 pathways. Autophagy.

[44] Sanchez-Alvarez R., De Francesco E.M., Fiorillo M., Sotgia F., Lisanti M.P. (2020). Mitochondrial Fission Factor (MFF) Inhibits Mitochondrial Metabolism and Reduces Breast Cancer Stem Cell (CSC) Activity. Front. Oncol..

[45] Tur J., Pereira-Lopes S., Vico T., Marin E.A., Munoz J.P., Hernandez-Alvarez M., Cardona P.J., Zorzano A., Lloberas J., Celada A. (2020). Mitofusin 2 in Macrophages Links Mitochondrial ROS Production, Cytokine Release, Phagocytosis, Autophagy, and Bactericidal Activity. Cell Rep..

[46] Kulkarni S.S., Joffraud M., Boutant M., Ratajczak J., Gao A.W., Maclachlan C., Hernandez-Alvarez M.I., Raymond F., Metairon S., Descombes P. (2016). Mfn1 Deficiency in the Liver Protects Against Diet-Induced Insulin Resistance and Enhances the Hypoglycemic Effect of Metformin. Diabetes.

[47] Sebastian D., Sorianello E., Segales J., Irazoki A., Ruiz-Bonilla V., Sala D., Planet E., Berenguer-Llergo A., Munoz J.P., Sanchez-Feutrie M. (2016). Mfn2 deficiency links age-related sarcopenia and impaired autophagy to activation of an adaptive mitophagy pathway. EMBO J..

[48] Song M., Dorn G.W. (2015). Mitoconfusion: Noncanonical functioning of dynamism factors in static mitochondria of the heart. Cell Metab..

[49] Yoon Y.S., Yoon D.S., Lim I.K., Yoon S.H., Chung H.Y., Rojo M., Malka F., Jou M.J., Martinou J.C., Yoon G. (2006). Formation of elongated giant mitochondria in DFO-induced cellular senescence: Involvement of enhanced fusion process through modulation of Fis1. J. Cell Physiol..

[50] Lee S., Jeong S.Y., Lim W.C., Kim S., Park Y.Y., Sun X., Youle R.J., Cho H. (2007). Mitochondrial fission and fusion mediators, hFis1 and OPA1, modulate cellular senescence. J. Biol. Chem..

[51] Park Y.Y., Lee S., Karbowski M., Neutzner A., Youle R.J., Cho H. (2010). Loss of MARCH5 mitochondrial E3 ubiquitin ligase induces cellular senescence through dynamin-related protein 1 and mitofusin 1. J. Cell Sci..

[52] Sabouny R., Shutt T.E. (2020). Reciprocal Regulation of Mitochondrial Fission and Fusion. Trends Biochem. Sci..

[53] Dorn G.W., Vega R.B., Kelly D.P. (2015). Mitochondrial biogenesis and dynamics in the developing and diseased heart. Genes. Dev..

[54] Shirihai O.S., Song M., Dorn G.W. (2015). How mitochondrial dynamism orchestrates mitophagy. Circ. Res..

[55] Ni H.M., Williams J.A., Ding W.X. (2015). Mitochondrial dynamics and mitochondrial quality control. Redox. Biol..

[56] Yu T., Robotham J.L., Yoon Y. (2006). Increased production of reactive oxygen species in hyperglycemic conditions requires dynamic change of mitochondrial morphology. Proc. Natl. Acad. Sci. USA.

[57] Wang W., Wang Y., Long J., Wang J., Haudek S.B., Overbeek P., Chang B.H., Schumacker P.T., Danesh F.R. (2012). Mitochondrial fission triggered by hyperglycemia is mediated by ROCK1 activation in podocytes and endothelial cells. Cell Metab..

[58] Parikh S.M., Yang Y., He L., Tang C., Zhan M., Dong Z. (2015). Mitochondrial function and disturbances in the septic kidney. Semin. Nephrol..

[59] Ayanga B.A., Badal S.S., Wang Y., Galvan D.L., Chang B.H., Schumacker P.T., Danesh F.R. (2016). Dynamin-Related Protein 1 Deficiency Improves Mitochondrial Fitness and Protects against Progression of Diabetic Nephropathy. J. Am. Soc. Nephrol.

[60] Galvan D.L., Long J., Green N., Chang B.H., Lin J.S., Schumacker P., Truong L.D., Overbeek P., Danesh F.R. (2019). Drp1S600 phosphorylation regulates mitochondrial fission and progression of nephropathy in diabetic mice. J. Clin. Investig..

[61] Guo K., Lu J., Huang Y., Wu M., Zhang L., Yu H., Zhang M., Bao Y., He J.C., Chen H. (2015). Protective role of PGC-1alpha in diabetic nephropathy is associated with the inhibition of ROS through mitochondrial dynamic remodeling. PLoS ONE.

[62] Liu X., Wang W., Song G., Wei X., Zeng Y., Han P., Wang D., Shao M., Wu J., Sun H. (2017). Astragaloside IV ameliorates diabetic nephropathy by modulating the mitochondrial quality control network. PLoS ONE.

[63] Coughlan M.T., Higgins G.C., Nguyen T.V., Penfold S.A., Thallas-Bonke V., Tan S.M., Ramm G., Van Bergen N.J., Henstridge D.C., Sourris K.C. (2016). Deficiency in Apoptosis-Inducing Factor Recapitulates Chronic Kidney Disease via Aberrant Mitochondrial Homeostasis. Diabetes.

[64] Yang Q., Dixit B., Wada J., Tian Y., Wallner E.I., Srivastva S.K., Kanwar Y.S. (2000). Identification of a renal-specific oxido-reductase in newborn diabetic mice. Proc. Natl. Acad. Sci. USA.

[65] Nayak B., Xie P., Akagi S., Yang Q., Sun L., Wada J., Thakur A., Danesh F.R., Chugh S.S., Kanwar Y.S. (2005). Modulation of renal-specific oxidoreductase/myo-inositol oxygenase by high-glucose ambience. Proc. Natl. Acad. Sci. USA.

[66] Zhan M., Usman I.M., Sun L., Kanwar Y.S. (2015). Disruption of renal tubular mitochondrial quality control by Myo-inositol oxygenase in diabetic kidney disease. J. Am. Soc. Nephrol..

[67] Hoppstadter J., Ammit A.J. (2019). Role of Dual-Specificity Phosphatase 1 in Glucocorticoid-Driven Anti-inflammatory Responses. Front. Immunol..

[68] Sheng J., Li H., Dai Q., Lu C., Xu M., Zhang J., Feng J. (2019). DUSP1 recuses diabetic nephropathy via repressing JNK-Mff-mitochondrial fission pathways. J. Cell Physiol..

[69] Migliaccio E., Giorgio M., Mele S., Pelicci G., Reboldi P., Pandolfi P.P., Lanfrancone L., Pelicci P.G. (1999). The p66shc adaptor protein controls oxidative stress response and life span in mammals. Nature.

[70] Sun L., Xiao L., Nie J., Liu F.Y., Ling G.H., Zhu X.J., Tang W.B., Chen W.C., Xia Y.C., Zhan M. (2010). p66Shc mediates high-glucose and angiotensin II-induced oxidative stress renal tubular injury via mitochondrial-dependent apoptotic pathway. Am. J. Physiol. Ren. Physiol..

[71] Zhan M., Usman I., Yu J., Ruan L., Bian X., Yang J., Yang S., Sun L., Kanwar Y.S. (2018). Perturbations in mitochondrial dynamics by p66Shc lead to renal tubular oxidative injury in human diabetic nephropathy. Clin. Sci..

[72] Sheng J., Li H., Dai Q., Lu C., Xu M., Zhang J., Feng J. (2018). NR4A1 Promotes Diabetic Nephropathy by Activating Mff-Mediated Mitochondrial Fission and Suppressing Parkin-Mediated Mitophagy. Cell Physiol. Biochem..

[73] Semenza G.L. (2003). Targeting HIF-1 for cancer therapy. Nat. Rev. Cancer.

[74] Ke Q., Costa M. (2006). Hypoxia-inducible factor-1 (HIF-1). Mol. Pharmacol..

[75] Jiang N., Zhao H., Han Y., Li L., Xiong S., Zeng L., Xiao Y., Wei L., Xiong X., Gao P. (2020). HIF-1alpha ameliorates tubular injury in diabetic nephropathy via HO-1-mediated control of mitochondrial dynamics. Cell Prolif..

[76] Sharfuddin A.A., Molitoris B.A. (2011). Pathophysiology of ischemic acute kidney injury. Nat. Rev. Nephrol..

[77] Funk J.A., Schnellmann R.G. (2012). Persistent disruption of mitochondrial homeostasis after acute kidney injury. Am. J. Physiol. Ren. Physiol..

[78] Liu J.X., Yang C., Zhang W.H., Su H.Y., Liu Z.J., Pan Q., Liu H.F. (2019). Disturbance of mitochondrial dynamics and mitophagy in sepsis-induced acute kidney injury. Life Sci..

[79] Lee S.Y., Kang J.M., Kim D.J., Park S.H., Jeong H.Y., Lee Y.H., Kim Y.G., Yang D.H., Lee S.H. (2017). PGC1alpha Activators Mitigate Diabetic Tubulopathy by Improving Mitochondrial Dynamics and Quality Control. J. Diabetes Res..

[80] Hickey F.B., Corcoran J.B., Griffin B., Bhreathnach U., Mortiboys H., Reid H.M., Andrews D., Byrne S., Furlong F., Martin F. (2014). IHG-1 increases mitochondrial fusion and bioenergetic function. Diabetes.

[81] Shenouda S.M., Widlansky M.E., Chen K., Xu G., Holbrook M., Tabit C.E., Hamburg N.M., Frame A.A., Caiano T.L., Kluge M.A. (2011). Altered mitochondrial dynamics contributes to endothelial dysfunction in diabetes mellitus. Circulation.

[82] Ma Y., Chen Z., Tao Y., Zhu J., Yang H., Liang W., Ding G. (2019). Increased mitochondrial fission of glomerular podocytes in diabetic nephropathy. Endocr. Connect..

[83] Lu B. (2009). Mitochondrial dynamics and neurodegeneration. Curr. Neurol. Neurosci. Rep..

[84] Wakabayashi T. (2002). Megamitochondria formation—physiology and pathology. J. Cell Mol. Med..

[85] Le T.H., Caldwell S.H., Redick J.A., Sheppard B.L., Davis C.A., Arseneau K.O., Iezzoni J.C., Hespenheide E.E., Al-Osaimi A., Peterson T.C. (2004). The zonal distribution of megamitochondria with crystalline inclusions in nonalcoholic steatohepatitis. Hepatology.

[86] Brown G.T., Kleiner D.E. (2016). Histopathology of nonalcoholic fatty liver disease and nonalcoholic steatohepatitis. Metabolism.

[87] Zottini M., Barizza E., Bastianelli F., Carimi F., Lo Schiavo F. (2006). Growth and senescence of Medicago truncatula cultured cells are associated with characteristic mitochondrial morphology. New Phytol..

[88] Clarkson M.R., Murphy M., Gupta S., Lambe T., Mackenzie H.S., Godson C., Martin F., Brady H.R. (2002). High glucose-altered gene expression in mesangial cells. Actin-regulatory protein gene expression is triggered by oxidative stress and cytoskeletal disassembly. J. Biol. Chem..

[89] Murphy M., Hickey F., Godson C. (2013). IHG-1 amplifies TGF-beta1 signalling and mitochondrial biogenesis and is increased in diabetic kidney disease. Curr. Opin. Nephrol. Hypertens..

[90] Murphy M., Docherty N.G., Griffin B., Howlin J., McArdle E., McMahon R., Schmid H., Kretzler M., Droguett A., Mezzano S. (2008). IHG-1 amplifies TGF-beta1 signaling and is increased in renal fibrosis. J. Am. Soc. Nephrol..

[91] Susin S.A., Lorenzo H.K., Zamzami N., Marzo I., Snow B.E., Brothers G.M., Mangion J., Jacotot E., Costantini P., Loeffler M. (1999). Molecular characterization of mitochondrial apoptosis-inducing factor. Nature.

[92] Vahsen N., Cande C., Briere J.J., Benit P., Joza N., Larochette N., Mastroberardino P.G., Pequignot M.O., Casares N., Lazar V. (2004). AIF deficiency compromises oxidative phosphorylation. EMBO J..

[93] Jeong H.Y., Kang J.M., Jun H.H., Kim D.J., Park S.H., Sung M.J., Heo J.H., Yang D.H., Lee S.H., Lee S.Y. (2018). Chloroquine and amodiaquine enhance AMPK phosphorylation and improve mitochondrial fragmentation in diabetic tubulopathy. Sci. Rep..

[94] Ni Z., Tao L., Xiaohui X., Zelin Z., Jiangang L., Zhao S., Weikang H., Hongchao X., Qiujing W., Xin L. (2017). Polydatin impairs mitochondria fitness and ameliorates podocyte injury by suppressing Drp1 expression. J. Cell Physiol..

[95] Shi S., Koya D., Kanasaki K. (2016). Dipeptidyl peptidase-4 and kidney fibrosis in diabetes. Fibrogenesis Tissue Repair.

[96] Zhang Q., He L., Dong Y., Fei Y., Wen J., Li X., Guan J., Liu F., Zhou T., Li Z. (2020). Sitagliptin ameliorates renal tubular injury in diabetic kidney disease via STAT3-dependent mitochondrial homeostasis through SDF-1alpha/CXCR4 pathway. FASEB J..

[97] Kubo A., Hidaka T., Nakayama M., Sasaki Y., Takagi M., Suzuki H., Suzuki Y. (2020). Protective effects of DPP-4 inhibitor on podocyte injury in glomerular diseases. BMC Nephrol..

[98] Cameron R.B., Gibbs W.S., Miller S.R., Dupre T.V., Megyesi J., Beeson C.C., Schnellmann R.G. (2019). Proximal Tubule beta 2-Adrenergic Receptor Mediates Formoterol-Induced Recovery of Mitochondrial and Renal Function after Ischemia-Reperfusion Injury. J. Pharmacol. Exp. Ther..

[99] Cleveland K.H., Brosius F.C., Schnellmann R.G. (2020). Regulation of mitochondrial dynamics and energetics in the diabetic renal proximal tubule by the beta2-adrenergic receptor agonist formoterol. Am. J. Physiol. Ren. Physiol..

[100] Yang S.K., Li A.M., Han Y.C., Peng C.H., Song N., Yang M., Zhan M., Zeng L.F., Song P.A., Zhang W. (2019). Mitochondria-Targeted Peptide SS31 Attenuates Renal Tubulointerstitial Injury via Inhibiting Mitochondrial Fission in Diabetic Mice. Oxid. Med. Cell Longev..

[101] Agil A., Chayah M., Visiedo L., Navarro-Alarcon M., Rodriguez Ferrer J.M., Tassi M., Reiter R.J., Fernandez-Vazquez G. (2020). Melatonin Improves Mitochondrial Dynamics and Function in the Kidney of Zucker Diabetic Fatty Rats. J. Clin. Med..

[102] Jiang K.F., Zhao G., Deng G.Z., Wu H.C., Yin N.N., Chen X.Y., Qiu C.W., Peng X.L. (2017). Polydatin ameliorates Staphylococcus aureus-induced mastitis in mice via inhibiting TLR2-mediated activation of the p38 MAPK/NF-kappaB pathway. Acta. Pharmacol. Sin..

[103] Gao Y., Zeng Z., Li T., Xu S., Wang X., Chen Z., Lin C. (2015). Polydatin Inhibits Mitochondrial Dysfunction in the Renal Tubular Epithelial Cells of a Rat Model of Sepsis-Induced Acute Kidney Injury. Anesth. Analg..

[104] Lee Y.H., Kim S.H., Kang J.M., Heo J.H., Kim D.J., Park S.H., Sung M., Kim J., Oh J., Yang D.H. (2019). Empagliflozin attenuates diabetic tubulopathy by improving mitochondrial fragmentation and autophagy. Am. J. Physiol. Ren. Physiol..

[105] Liu X., Xu C., Xu L., Li X., Sun H., Xue M., Li T., Yu X., Sun B., Chen L. (2020). Empagliflozin improves diabetic renal tubular injury by alleviating mitochondrial fission via AMPK/SP1/PGAM5 pathway. Metabolism.

[106] Yin J., Xing H., Ye J. (2008). Efficacy of berberine in patients with type 2 diabetes mellitus. Metabolism.

[107] Qin X., Zhao Y., Gong J., Huang W., Su H., Yuan F., Fang K., Wang D., Li J., Zou X. (2019). Berberine Protects Glomerular Podocytes via Inhibiting Drp1-Mediated Mitochondrial Fission and Dysfunction. Theranostics.

